# The Protective Role of Sulfated Polysaccharides from Green Seaweed *Udotea flabellum* in Cells Exposed to Oxidative Damage

**DOI:** 10.3390/md16040135

**Published:** 2018-04-20

**Authors:** Fernando Bastos Presa, Maxsuell Lucas Mendes Marques, Rony Lucas Silva Viana, Leonardo Thiago Duarte Barreto Nobre, Leandro Silva Costa, Hugo Alexandre Oliveira Rocha

**Affiliations:** 1 Departamento de Bioquímica, Universidade Federal do Rio Grande do Norte, Natal 59078-970, Rio Grande do Norte, Brazil; fernandobpresa@gmail.com (F.B.P.); maxsuell_lucas@hotmail.com (M.L.M.M.); rony_lucas@hotmail.com (R.L.S.V.); leo.dnobre@gmail.com (L.T.D.B.N.); 2 Programa de Pós-graduação em Ciências da Saúde, Universidade Federal do Rio Grande do Norte, Natal 59078-970, Rio Grande do Norte, Brazil; 3 Instituto Federal de Educação, Ciência, e Tecnologia do Rio Grande do Norte (IFRN), Ceara-Mirim 59900-000, Rio Grande do Norte, Brazil; leandro-silva-costa@hotmail.com

**Keywords:** sulfated galactan, 3T3 fibroblasts, green seaweed

## Abstract

Seaweed is a rich source of bioactive sulfated polysaccharides. We obtained six sulfated polysaccharide-rich fractions (UF-0.3, UF-0.5, UF-0.6, UF-0.7, UF-1.0, and UF-2.0) from the green seaweed *Udotea flabellum* (UF) by proteolytic digestion followed by sequential acetone precipitation. Biochemical analysis of these fractions showed that they were enriched with sulfated galactans. The viability and proliferative capacity of 3T3 fibroblasts exposed to FeSO_4_ (2 µM), CuSO_4_ (1 µM) or ascorbate (2 mM) was not affected. However, these cells were exposed to oxidative stress in the presence of FeSO_4_ or CuSO_4_ and ascorbate, which caused the activation of caspase-3 and caspase-9, resulting in apoptosis of the cells. We also observed increased lipid peroxidation, evaluated by the detection of malondialdehyde and decreased glutathione and superoxide dismutase levels. Treating the cells with the ultrafiltrate fractions (UF) fractions protected the cells from the oxidative damage caused by the two salts and ascorbate. The most effective protection against the oxidative damage caused by iron was provided by UF-0.7 (1.0 mg/mL); on treatment with UF-0.7, cell viability was 55%. In the case of copper, cell viability on treatment with UF-0.7 was ~80%, but the most effective fraction in this model was UF-2.0, with cell viability of more than 90%. The fractions, mainly UF-0.7 and UF-2.0, showed low iron chelating activity, but high copper chelating activity and total antioxidant capacity (TAC). These results suggested that some of their protective mechanisms stem from these properties.

## 1. Introduction

Copper and iron are two of the most abundant metals in the human body. They play several important roles in cellular metabolism. Copper is an essential micronutrient in human nutrition; it is a component of metalloenzymes and acts as an electron donor or acceptor, while iron forms complexes with proteins, such as flavin-iron enzymes, transferrin, ferritin, myoglobin or hemoglobin (responsible for oxygen transport), and with enzymes involved in electron transfer and oxidation-reduction reactions [1]. However, high levels of these transition metal ions in the form Cu^2+^ and Fe^2+^ are responsible for the production of reactive oxygen species (ROS), which leads to oxidative stress in cells, causing cellular damage and diseases. High levels of copper in the body, either by ingestion or genetic predisposition (Wilson’s disease and Menkes syndrome), may contribute to mitochondrial dysfunction, subsequently leading to the production of ROS and induction of apoptosis. Consequently, several organs may suffer from its toxic effects [2]. An excess of iron in the body results in disorders, such as cirrhosis, liver cancer, diabetes, arrhythmia, and osteoporosis [3]. The use of chelating agents is always recommended in case of excess of physiological copper or iron [4]. However, these agents have several side effects [5]; thus, there is a continued search for new chelating agents.

Campo and colleagues [6] demonstrated that glycosaminoglycans, a group of sulfated polysaccharides (SPs), have antioxidant activity and can chelate both Cu^++^ and Fe^++^, indicating that these compounds may serve as prospective drugs to combat the damaging effects of copper and iron. However, glycosaminoglycans are found in small amounts in all their sources, which could make their commercial use difficult. Other SPs are found in all seaweeds, and in some species of animals, plants, bacteria, fungi, and other microorganisms [7]. However, only seaweeds can synthetize SPs in large quantities. In addition, several SPs from seaweeds showed iron chelation activity, which makes seaweeds potential sources of chelating SPs.

Ulvans, green seaweed-origin SPs from the genus *Ulva*, showed antioxidant activity in several in vitro models [8]. In the present study, we draw attention to the SPs from *Ulva pertusa*, as they are known to form chelates with iron [9]. We have previously demonstrated that SPs from tropical green seaweeds, namely *Caulerpa cupressoides* [10], *Caulerpa prolifera* and *Caulerpa sertularioides* [11], have iron chelation activity.

Green seaweeds synthesize a range of sulfated homo- [12] and heteropolysaccharides [13]. These SPs have distinct chemistries and functions without parallelism in other organisms. This uniqueness results in the low degree of redundancy in the structure and mechanism of action of SPs, which has in turn resulted in renewed interest in the recognition of green seaweeds as novel sources of SPs [14]. However, there are fewer studies on SPs of green algae compared to those on other types of seaweed. 

Recently, we found that an extract enriched in SPs from another tropical seaweed, *Udotea flabellum*, showed copper chelating activity (unpublished data). This seaweed is found easily on the Brazilian coast, and antioxidative properties of polysaccharides from its extracts have not been evaluated to date. Therefore, the aim of this study was to obtain SP-rich fractions from *U. flabellum* and evaluate their potential as protective agents in two models of oxidative stress caused by the presence of copper or iron.

## 2. Results

### 2.1. Chemical Analyses

We used a low-cost, widely reproducible method, which combined proteolysis and sequential acetone precipitation, to obtain six sulfated polysaccharide-rich fractions (named UF-0.3, UF-0.5, UF-0.6, UF-0.7, UF-1.0, and UF-2.0) from the green seaweed *U*. *flabellum*. The results of the chemical analysis of the fractions and their polysaccharide yields are summarized in Table 1. The yield ranged from 4.2% (UF-0.3) to 36.9% (UF-0.5); UF-0.3 and UF-1.0 were contaminated with proteins. The sulfate percentage indicated that it was present in all fractions.

**Figure 1:**
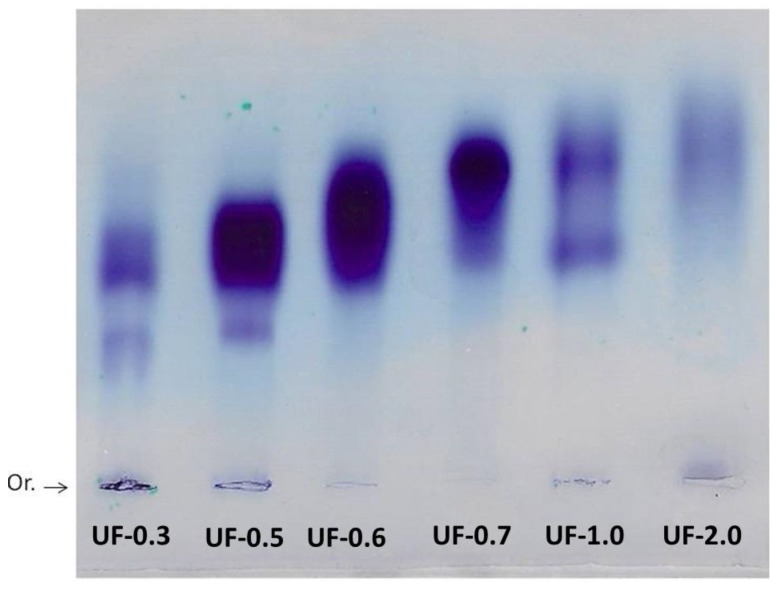
Electrophoresis in 0.05 M diaminopropane acetate buffer, pH 9.0, of fractions obtained by acetone precipitation. About 5 µL (50 µg) of each polysaccharide was applied in agarose gel prepared in diaminopropane acetate buffer and subjected to electrophoresis, as described in methods. Or.—origin.

In order to confirm whether the sulfate was covalently linked to polysaccharides, we subjected the fractions to agarose gel electrophoresis. After the gel was stained with toluidine blue (Figure 1) we found that all fractions contained electrophoretically mobile purple colored bands, characteristic to sulfated polysaccharides. We observed slight differences in the mobility of the bands of each fraction, and multiple bands were observed for some fractions (UF-0.3 and UF-1.0).

Table 1 also shows that galactose and glucose are present in all fractions, but in different proportions. Galactose was found to be the predominant sugar in all fractions; therefore, it was chosen as a reference to determine the proportion of other sugars. Glucose was found in large proportions in the fractions UF-0.3, UF-1.0, and UF-2.0. Mannose was not detected in UF-0.3, and its percentage increased with respect to other sugars in subsequent fractions reaching the maximum in UF-2.0. Xylose was absent in fractions UF-0.3 and UF-2.0.

### 2.2. Effect of Sulfated Polisaccharides on Cell Viability and Cell Proliferation

**Figure 2:**
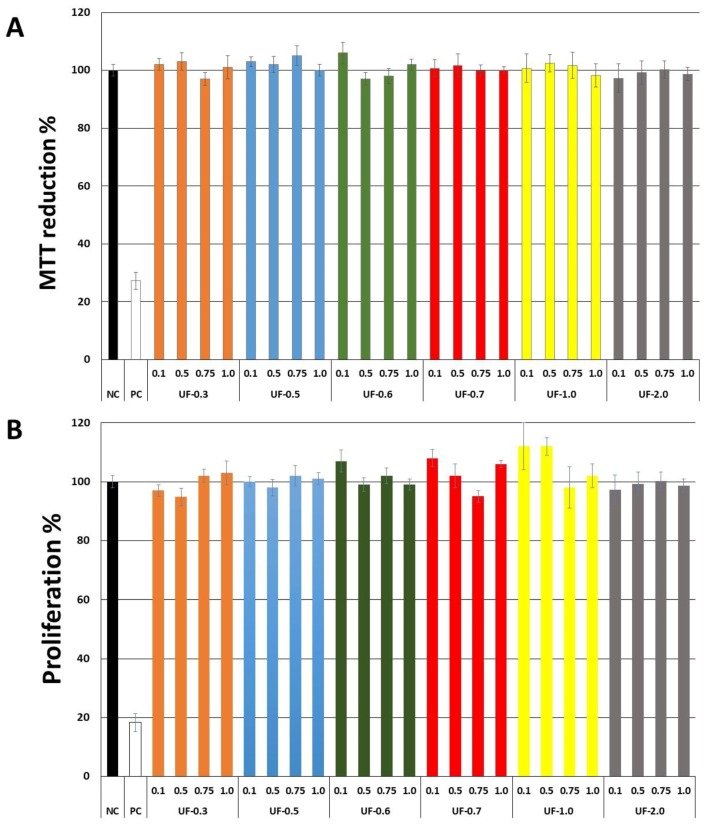
Effect of different concentrations (mg/mL) of sulfated polysaccharides from *U. flabellum* on the ability of fibroblast (3T3) cells to reduce 3-(4,5-dimethylthiazol-2-yl)-2,5-diphenyltetrazolium bromide (MTT) (**A**) and incorporate 5-bromo-2-deoxyuridine (BrdU) BrdU (**B**). NC—negative control composed only of culture medium with fetal bovine serum. PC—positive control-culture medium with fetal bovine serum and cisplatin (2 µg/mL).

Initially, we evaluated whether sulfated polysaccharides were toxic to 3T3 fibroblast cells. As seen in Figure 2A, the polysaccharides, at the proportions determined previously, failed to have any effect on the ability of fibroblasts to reduce 3-(4,5-dimethylthiazol-2-yl)-2,5-diphenyltetrazolium bromide (MTT). In contrast, treatment with cisplatin, which was used as a positive control, caused 3T3 cells to reduce only 27% of the MTT molecules.

Figure 2B shows that the sulfated polysaccharides also did not affect the proliferation of 3T3 cells, as the incorporation of 5-bromo-2-deoxyuridine (BrdU) by the cells incubated in the presence of fractions was similar with the cells of the negative control group (cells exposed only to medium and fetal bovine serum).

### 2.3. Evaluation of SP Chelation Activity

As UF-0.3 and UF-1.0 showed protein contamination, we did not use these fractions in subsequent experiments. 

**Figure 3:**
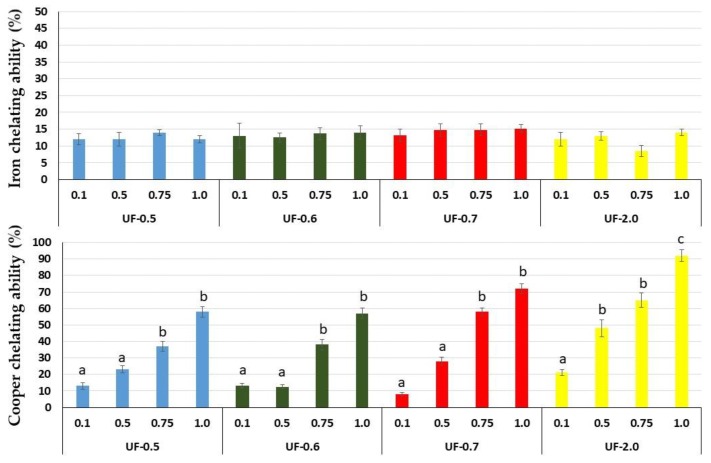
Copper- and iron-chelating ability of sulfated polysaccharides from *U. flabellum.* Data are presented as means ± standard deviation. Different lowercase letters indicate a significant difference (*p* < 0.05) between the ability of polysaccharides to chelate copper or iron. Ethylenediaminetetraacetic acid (EDTA) was used as the positive control, which corresponded to 100% chelating ability (maximum chelating ability was 0.025 and 0.02 mg/mL in the copper and iron analyses, respectively).

As seen in Figure 3, all samples had low iron chelating activity (<20%) compared to that of ethylenediaminetetraacetic acid (EDTA), used as the positive control. In contrast, the polysaccharides showed a dose-dependent effect when copper was used. The most significant chelation (~90%) was observed for UF-2.0.

### 2.4. Effect of Sulfated Polysaccharides on MTT Reduction in 3T3 Fibroblast Cells

**Figure 4:**
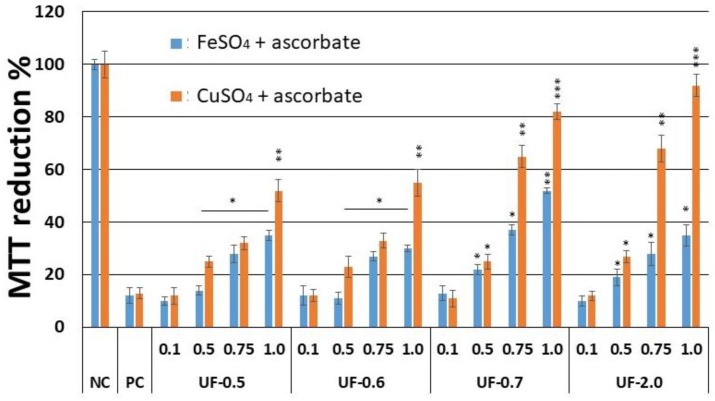
Effect of different amounts (mg/mL) of sulfated polysaccharides from *U. flabellum* on the ability of fibroblast (3T3) cells to reduce MTT in the two models of oxidative stress. NC—negative control composed only of culture medium with fetal bovine serum. PC—positive control-culture medium with fetal bovine serum, FeSO_4_ or CuSO_4,_ and ascorbate. *** *p* < 0.001; ** *p* < 0.01; * *p* < 0.05 vs. PC.

As Figure 4 shows, the sulfated polysaccharides protected the cells from the action of FeSO_4_ and CuSO_4_ -. On using FeSO_4_, UF-0.7 was the only fraction that caused the 3T3 cells to reduce more than 50% of the MTT molecules that were present in the culture medium. In contrast, when we used CuSO_4_, the cells that were subjected to the higher concentrations of samples were able to reduce more than 50% of the MTT molecules. Notably, fractions UF-0.7 and UF-2.0, (1.0 mg/mL) enabled cells to reduce ~81% and ~95% of the MTT molecules, respectively.

We also exposed the 3T3 cells to FeSO_4_ (2 µM), CuSO_4_ (1 µM) or ascorbate (2 mM) and we observed that the ability of 3T3 cells to reduce MTT was not affected in any of the conditions evaluated.

### 2.5. Effect of Sulfated Polysaccharides on 3T3 Fibroblast Caspase-3 and Caspase-9

**Figure 5:**
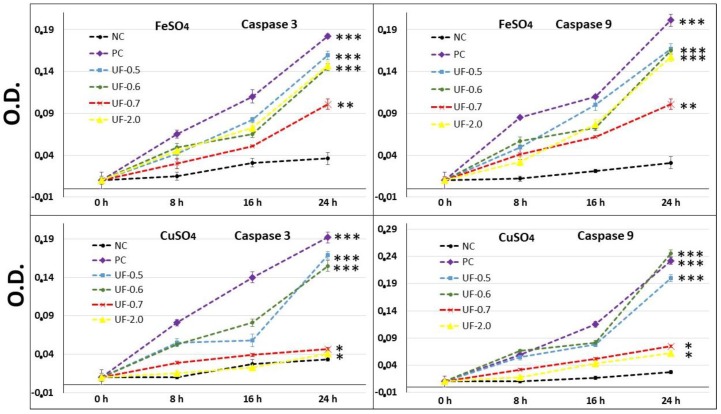
Effect of different amounts (mg/mL) of sulfated polysaccharides from *U. flabellum* on caspase-3 and caspase-9 activity in 3T3 cells exposed to two models of oxidative stress. O.D.—optical density. NC—negative control composed only of culture medium with fetal bovine serum. PC—positive control-culture medium with fetal bovine serum FeSO_4_ or CuSO_4_ and ascorbate. *** *p* < 0.001; ** *p* < 0.01; * *p* < 0.05 vs. NC.

Several studies show that FeSO_4_ and CuSO_4_ induce cell death along with ascorbate by promoting the activation of caspases. Therefore, we evaluated the activity of capase-3 and caspase-9 from the 3T3 fibroblasts that were exposed to FeSO_4_ and CuSO_4_, in the absence and presence of sulfated polysaccharides (1 mg/mL). Figure 5 shows that the activity of caspase-3 and caspase-9 is very high after the cells have been exposed to oxidizing conditions positive control (PC), metal plus ascorbate). Moreover, the presence of polysaccharides decreased the activity of both caspases in various conditions. However, polysaccharides were less effective when the cells were exposed to FeSO_4_ than when exposed to CuSO_4_.

When the effect of each fraction was studied individually, the activity of the caspases in fractions UF-0.5, UF-0.6, and UF-2.0 in the presence of FeSO_4_ was about 25% lower than that observed with the PC group. On the other hand, the activity of both caspases corresponded to about 50% to that observed in the PC group.

### 2.6. Lipid Peroxidation Analysis

Estimation of malondialdehyde levels was performed to determine the degree of free radical production in 3T3 cells (Table 2). The concentration of malondialdehyde found in the cells of the control group was approximately 250 mmol/10^6^ cells, and this was considered to be the physiological concentration. These levels were about ten times higher in the presence of metals and ascorbate. This indicates that the presence of these compounds promoted oxidative damage.

When the cells were exposed to FeSO_4_ or CuSO_4_ and ascorbate along with the polysaccharides the levels of malondialdehyde were significantly lower than those observed in the PC group. The most efficient samples to reduce malondialdehyde levels were UF-0.7 and UF-2.0.

### 2.7. Assessment of the Cell Antioxidant Status

The concentration of glutathione (GSH) and Superoxide dismutase (SOD) were assayed in order to evaluate antioxidant balance after free radical production (Table 2). The levels of GSH (6.78 ± 2.21 mmol/10^6^ cell) and SOD (33.5 ± 1.1 mmol/10^6^ cell) found in the control cells were considered physiological. A significant reduction in levels of both antioxidants was observed in several experimental conditions, and this effect was more pronounced in the PC group.

When cells were treated with copper, ascorbate, and polysaccharides, we found no significant difference in GSH levels between the treated groups and the negative group (NC) group. When cells were exposed to iron, similar results were obtained with UF-0.7 and UF-2.0. This shows that these two fractions prevented a decrease in the levels of GSH even when the cells were subjected to oxidative damage.

The protective effect of polysaccharides was less pronounced in the case of SOD. However, treatment with both UF-0.7 and UF-2.0, in the presence of iron or copper, showed decreased levels of this enzyme

### 2.8. In Vitro Evaluation of Antioxidant Capacity of Polysaccharides

**Figure 6:**
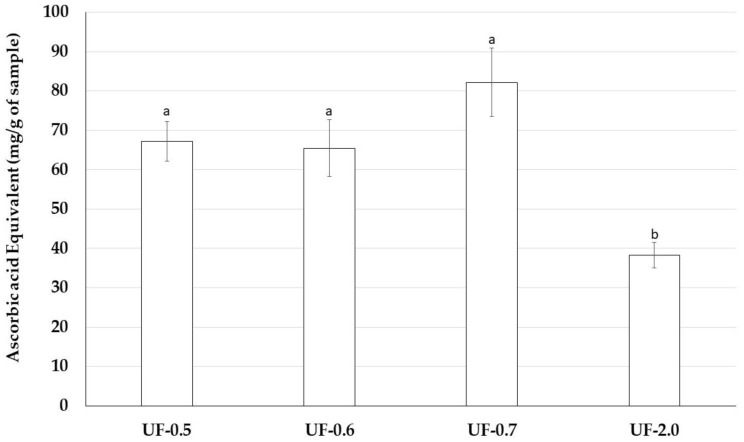
Total antioxidant capacity of sulfated polysaccharides-rich fractions from *U. flabellum.* Data are presented as means ± standard deviation. ^a, b^ Different lowercase letters indicate a significant difference (*p* < 0.05) between the total antioxidant capacities of samples.

The total antioxidant capacity (TAC) test aims to evaluate the ability of a compound to donate electrons to another molecule, which stabilizes it. UF-2.0 presented significantly lower TAC than the other samples. The other samples presented significantly similar TAC as shown in Figure 6.

The polysaccharides at all concentrations tested (from 0.1 to 1.0 mg/mL) did not exhibit any hydroxyl radical scavenging activity. In addition, only UF-2.0 exhibited superoxide radical scavenging activity, but this activity was 15%, and it was only detected at the highest concentration (1.0 mg/mL).

The last antioxidant evaluation test we performed was to determine the reducing power of the samples. UF-0.5 and UF-2.0 showed activity (~35%), but we only detected this activity at the highest concentration (1.0 mg/mL).

## 3. Discussion

The data presented in Table 1 indicate that all fractions contained polysaccharides and sulfate. The confirmation that sulfate ions were covalently bound to the polysaccharides was achieved by using agarose gel electrophoresis, as the free sulfate ions and small sulfated molecules would not be retained in the gel mesh [15]. Based on these data, we obtained six sulfated polysaccharide-rich fractions from *U. flabellum*.

We obtained the fractions using differential precipitation with acetone, which was due to different interaction forces between polysaccharide molecules with water. Therefore, the addition of increasing volumes of acetone to the samples is necessary during precipitation. The polysaccharides, which are most weakly bound, precipitate with lower concentrations of acetone and those, which are more strongly bound, requiring higher concentrations of acetone for precipitation.

Agarose gel electrophoresis allowed the visualization and verification of the existence of sulfated polysaccharides in all fractions. The purple color obtained is typical of SPs due to the interaction of the toluidine blue dye with the sulfated group present in the polysaccharides [15].

Generally, in agarose gel electrophoresis systems, the mobility of the polysaccharides depends on their charges; for example, the more negative ones have greater mobility than the negative ones. However, as suggested by Dietrich and Dietrich [15], the use of 1,3-diaminopropane was important for the visualization of different polysaccharide molecules in the fractions. In the pH of the test (9.0) the sulfated polysaccharides assume a conformation in which some sulfate groups are exposed and others not. These exposed sulfates react with the diamine and their negative charges are quenched. This will induce a change in the conformation of the polysaccharide, and new sulfate groups are exposed and, accordingly, will react with the diamine. This process continues until equilibrium is reached, and the conformation of the polysaccharide no longer changes. However, the polysaccharide, in this conformational equilibrium condition, will still have sulfate groups that did not react with the diamine, and it will be these groups that will permit the mobility of the polysaccharide in the agarose during the electrophoresis. In short, polysaccharide molecules that have the same structure will have the same interaction with the diamine, and consequently have the same electrophoretic motility; whereas, structurally different polysaccharides have different electrophoretic mobilities [15]. In Figure 2, the presence of multiple bands with different mobilities in some fractions indicated that there are at least two polysaccharides in these Udotea fractions.

The results discussed so far (acetone precipitation, electrophoresis, and monosaccharide composition) show that *U. flabellum* synthesizes more than one type of sulfated polysaccharide. This agrees with reports that describe green seaweeds synthesizing more than one type of SP [13].

The analysis of the monosaccharide composition showed that all the fractions are heterogeneous, and that their main constituent is galactose. The predominance of one or more monomers over others as observed in the fractions of the *U. flabellum* was not surprising, as there are previous reports with data from other seaweeds, supporting a similar predominance [16,17]. Moreover, galactose has been described as the main component of SPs in other seaweeds, such as *Caulerpa cupressoides* [10] and *Codium isthmocladum* [12].

Our first objective was to rule out the toxicity of *Udotea* SPs to fibroblast 3T3 cells. As seen in Figure 2, *Udotea* SPs were not cytotoxic, and did not influence the proliferation of fibroblasts. Therefore, we hypothesized these SPs to be putative protective agents under conditions of oxidative stress. Two samples (UF-0.3 and UF-1.0) were not used due to protein contamination.

Production of reactive oxygen species leads to oxidative stress in cells, and, consequently, are harmful to organisms. In many oxidative stress models, one of the first reactive species of oxygen that is formed in great quantity is the superoxide anion. It is considered a reactive primary species, as it can generate reactive derivatives by direct interactions with other molecules or by processes catalyzed by metals or enzymes. In addition, under conditions of stress, superoxide anions can interact with proteins with iron as a cofactor and release iron groups from them [18]. Superoxide anion is a substrate for SOD that transforms it into hydrogen peroxide. These two species have a short-lived action. However, they can react with iron or copper through the Fenton and Haber-Weiss reactions, which leads to formation of the hydroxyl radical (OH•), which is a much more damaging ROS [19].

Several studies have demonstrated that the presence of iron or copper sulfate under culture conditions promotes oxidative stress. In addition, the presence of ascorbate also contributes to enhance the production of the detrimental OH• [6,20]. Therefore, we chose the model of cellular damage in the presence of these two salts and ascorbate to evaluate the protective action of Udotea SPs.

Because the oxidation of a substrate is caused by a reactive species in a chain reaction that includes three steps (initiation, propagation, and termination), the action of the antioxidants is evaluated through several mechanisms. Chelating agents act in both the initiation and propagation steps. Thus, they are antioxidant compounds because they prevent the formation of radicals and thus indirectly reduce the risk posed by radicals [9].

That is why we evaluated the chelating capacity of *Udotea* SPs. All SPs showed high copper chelating activity, with emphasis on UF-0.7 and UF-2.0 at 1 mg/mL, which presented activities superior to 80% and 90%, respectively. SPs from the brown seaweed *Undaria pinnatifida* also showed high copper chelating activity, but this activity was obtained only with high concentration (2.5 mg/mL) [21]. SPs from *Dictyopteris justii* (brown seaweed) also showed iron chelating activity, and one of these polysaccharides had a maximum activity of ~80% (1 mg/mL) [22]. We have not identified other reports that have evaluated the copper chelating activity of SPs from seaweeds. Many reports show a positive correlation between the activity of SPs and their sulfate content. We did not identify this correlation with *Udotea* SPs. Indeed, the copper chelating activity of polysaccharides appears to be more related to the conformation of the polysaccharide than to the presence of negatively charged groups, since neutral polysaccharides showed this activity. For example, yeast-neutral polysaccharides showed 80% activity when evaluated at concentrations similar to those used in the present study [23].

This copper chelating activity of *Udotea* SPS was reflected in cell-based assays. We found that UF-0.7 and UF-2.0, which were the most potent chelators, were also the ones that most protected the cells from damage caused by oxidative stress. Moreover, as shown in Figure 3, this activity was dose dependent. UF-1.0 contained the most potent SPs, which was demonstrated when SPs were used at similar concentrations (Figure 4 and Figure 5, and Table 2).

Campo and colleagues [6] showed that the glycosaminoglycans, hyaluronic acid and chondroitin-4-sulfate, protect fibroblastic cells against the damage caused by the presence of CuSO_4_ and ascorbate. However, when we performed the cytotoxicity assay (Figure 4.), the rate of reduction of MTT was 80%, and this was only achieved at a concentration of 2.0 mg/mL, which is twice the concentration used with UF-2.0. In addition, with UF-2.0, we achieved an MTT reduction rate of over 90%.

Our results showed that when 3T3 cells were exposed to CuSO_4_, the levels of caspase-3 (executioner) and caspase-9 (initiator) in the cells were very high, corroborating with the MTT test data (Figure 5). This implies that the cells under conditions of oxidative damage tend to die by apoptosis. Caspases are a group of proteases important for cellular apoptosis to occur. There are initiator caspases and executioner caspases, which are activated by the initiator caspases. The activation of caspases under conditions of oxidative stress and their involvement in the process of cell death induced by it have been reported since a long time [24]. The activity levels of these caspases in the groups of cells treated with UF-0.7 and UF-2.0 were similar to those observed in the NC group. This data demonstrated that fractions UF-0.7 and UF-2.0 could act as chelating agents, thus decreasing the oxidative stress, activation of caspases, and the number of dead cells (as seen indirectly via the MTT test).

To confirm that the oxidative stress in the cells was reduced by the presence of sulfated polysaccharides, we evaluated the levels of three markers of oxidative stress SOD, GSH, and malondialdehyde. 

Free radical-mediated cell damage can generally cause lipid peroxidation, and malondialdehyde levels are a good marker of this event, the higher the amount of this molecule, the greater the lipid peroxidation [6]. The presence of sulfated polysaccharides prevented the levels of malondialdehyde from getting so high. In fractions UF-0.7 and UF-2.0, these levels were only 5 times higher than those observed in the PC.

The concentration of SOD and GSH decreases considerably when cells are exposed to oxidative damage. This is because SOD and GSH are consumed by the reactive species that are formed [25]. 

This explains the decrease in the levels of SOD and GSH when 3T3 cells are exposed to oxidative damage. In both cases, we observed that the sulfated polysaccharides, mainly UF-0.7 and UF-2.0, protected the cells against the oxidative damage.

The mechanism by which SPs from *Udotea* reduce cellular damage against free radical overproduction is similar to that of hyaluronic acid and chondroitin-4-sulfate, typical SPs that can behave as chelating agents [6,26]. We had hypothesized that the protective action of polysaccharides from this seaweed against oxidative damage occurs mainly because of its copper chelating properties.

*Udotea* SPs presented low iron chelating activity. However, their presence protected 3T3 cells against oxidative damage caused by exposure to FeSO_4_. This was observed in all the tests performed. In addition, the data showed that, unlike in the CuSO_4_ assays, the most effective polysaccharide was UF-0.7. These data indicated that oxidative stress caused by iron was attenuated by the polysaccharides via other mechanisms besides chelation to wield their antioxidant action, consequently, protecting the cell against oxidative damage.

Therefore, we assessed antioxidant properties of *Udotea* SPs using five antioxidant tests. *Udotea* SPs were effective only in the TAC. In this test, we verified that the activity of UF-2.0 was lower than that of the other SPs. In contrast, UF-0.7 was the most potent antioxidant among all SPs. In the TAC assay, the sample is evaluated as an electron donor; the higher the value obtained in the TAC, the greater its ability to donate electrons and, consequently, to neutralize the ROS.

TAC data may partly explain why *Udotea* SPs, although not strong iron chelators, are able to protect cells from the oxidative stress caused by the presence of iron. We are interested in identifying other mechanisms that allow the *Udotea* SPs to act as protectors of cells exposed to oxidative stress caused by iron.

## 4. Materials and Methods

### 4.1. Materials

Iron (II) sulfate, potassium ferricianyde, sulfuric acid and acetonitrile were obtained from Merck (Darmstadt, Germany). Nitro Blue Tetrazolium (NBT), monosaccharides, methionine and ammonium molybdate were purchased from Sigma-Aldrich Co. (St. Louis, MO, USA). Cell culture medium components (Dulbecco’s Modified Eagle Medium-DMEM), trypsin and newborn calf serum (FCS) were obtained from Cultilab (Campinas, Brazil). l-glutamine, sodium bicarbonate, sodium pyruvate and phosphate buffered saline (PBS) were purchased from Invitrogen Corporation (Burlington, ON, USA). All other solvents and chemicals were of analytical grade. 

Eukaryotic cells—Mouse embryonic fibroblast cells (NIH/3T3 ATCC^®^ CRL-1658™-3T3, Manassas, VA, USA) were grown in Dulbecco’s modified Eagle’s medium (DMEM) with 10% of fetal bovine serum (FBS), 10 mg/mL streptomycin, and 10,000 IU penicillin.

### 4.2. Extraction of Sulfated Polysaccharide-Rich Fractions

The seaweed *Udotea flabellum* was collected at Búzios Beach, Nísia Floresta-RN, Brazil. The alga was stored in our laboratory and dried at 50 °C under ventilation in an oven, ground in a blender and incubated with ethanol to eliminate lipids and pigments. About 90 g of powdered alga was suspended with five volumes of 0.25 M NaCl and the pH was adjusted to 8.0 with NaOH. Next, 900 mg of Prolav 750 (Prozyn Biosolutions, São Paulo, SP, Brazil), a mixture of alkaline proteases, was added for proteolytic digestion. After incubation for 24 h at 60 °C under agitation and periodical pH adjustments, the mixture was filtered through cheesecloth. The filtrate was fractionated by precipitation with acetone as follows: 0.3 volumes of ice-cold acetone was added to the solution under gentle agitation and maintained at 4 °C for 24 h. The precipitate formed was collected by centrifugation (10,000× *g*, 20 min), vacuum dried, resuspended in distilled water, and analyzed. The operation was repeated by adding 0.5, 0.6, 0.7, 1.0, and 2.0 volumes of acetone to the supernatant.

### 4.3. Chemical Analysis and Monosaccharide Composition

Sulfate content was determined according to the gelatin-barium method [27], using sodium sulfate (1 mg/mL) as standard and after acid hydrolysis of the polysaccharides (4 M HCl, 100 °C, 6 h). Protein content was measured using Spector’s method [28]. The polysaccharides were hydrolyzed with 0.5, 1, 2, and 4 M, respectively, for various lengths of time, (0.5, 1, 2 and 4 h), at 100 °C. Reducing sugars were determined using the Somogyi-Nelson method [29]. After acid hydrolysis, sugar composition was determined by a LaChrom Elite^®^ HPLC system from VWR-Hitachi (Hitachi, Ltd., Tokyo, Japan) with a refractive index detector (RI detector model L-2490). A LichroCART^®^ 250-4 column (250 mm × 40 mm) packed with Lichrospher^®^ 100 NH2 (5 µm) was coupled to the system. The sample mass used was 0.2 mg and analysis time was 25 min. The following sugars were analyzed as references: arabinose, fructose, fucose, galactose, glucose, glucosamine, glucuronic acid, mannose, and xylose.

### 4.4. 3-(4,5-dimethylthiazol-2-yl)-2,5-diphenyltetrazolium Bromide Test

For the tests, 0.5 × 10^4^ cells were grown in 96-well plates with DMEM medium containing the samples in concentrations of 0.1, 0.5, 0.75 and 1 mg/mL for 24 h (each concentration in triplicate). The cell capacity to reduce MTT was determined by the colorimetric test of MTT as described earlier [30].

### 4.5. 5-bromo-2-deoxyuridine Incorporation

The cells (5 × 10^3^ cells/well) were seeded into 96-well plates with 300 μL of fresh medium and incubated for 12 h at 37 °C and 5.0% CO_2_. The medium was removed, the samples in DMEM medium was added to a final concentration between 0.1 and 1.0 mg/mL, and plates were incubated for 24 h, at 37 °C and 5.0% CO_2_. After incubation, unbound samples were removed by washing the cells twice with 200 μL PBS and BrdU incorporation was determined according to manufacturer’s instruction (BrdU cell proliferation assay kit-Cell Signaling, Danvers, MA, USA). 

### 4.6. Induced Oxidative Stress Assay

3T3 cells (1 × 10^6^ cells/mL) were placed in 6-well plates in de presence of 1 mL of DMEM supplemented with 10% FCS. After 24 h, the plates were washed and 1 mL of DMEM supplemented with 10% FCS and sulfated polysaccharides (0.1; 0.5; 0.75 or 1.0 mg/mL), CuSO_4_ (1 µM, final concentration) or FeSO_4_ (2 µM, final concentration) were added. 15 min after, 10 µL of 200 mM ascorbate acid (2 mM final concentration) was added. The plates were kept in culture condition (37 °C; 5% CO_2_; dark) for 90 min; thus, the medium was replaced by 1 mL of the same fresh medium. After 24 h, the cells were submitted to MTT test as described above.

### 4.7. Caspase-3 and -9 Activity Assays

3T3 cells (1 × 10^6^ cells/mL) were placed in 6-well plates in de presence of 1 mL of DMEM supplemented with 10% FCS. After 24 h, the plates were washed and 1 mL of DMEM supplemented with 10% FCS, FeSO_4_, CuSO_4_, ascorbate and/or sulfated polysaccharides (1 mg/mL) was added. After 90 min, the medium was replaced by fresh medium. 8, 16 and 24 h the plates were washed with ice-cold PBS and scraped into 200 mL lysis buffer (50 mM Tris-HCl (pH 7.4), 1% Tween 20, 0.25% sodium deoxycholate, 150 mM NaCl, 1 mM EDTA, 1 mM Na3VO4, 1 mM NaF], and protease inhibitors (1 mg/mL aprotinin, 10 mg/mL leupeptin and 1 mM 4-(2-aminoethyl) benzenesulfonyl fluoride) for 2 h in ice. The same conditions were used for untreated cells in the 0, 8, 16 and 24 h. Protein extracts were cleared by centrifugation and protein concentrations were determined using Bradford reagent [28] with bovine serum albumin as standard. In vitro caspase-3 and -9 protease activity was measured using a caspase activation kit according to the manufacturer’s protocol (Invitrogen, São Paulo, Brazil). For this, 50 µL of cell lysate was mixed with 50 µL of 2x reaction buffer (containing 10 µL of 1 M dithiothreitol and 5 µL of 4 mM synthetic tetrapeptide Asp-Glue-Val (for caspase 3) or Leu-Glu-His-Asp (for caspase 9) conjugated top-nitroanilide (pNA)) in a 96-well plate, after which the mixture was incubated for 2 h at 37 °C in the dark. Active caspase cleaves the peptide and releases the chromophore pNA that can be detected spectrophotometrically at a wavelength of 405 nm. Theoretically, the apoptotic cell lysates containing active tested caspases should yield a considerable emission compared with the non-apoptotic cell lysates. Data presented are representative of those obtained in at least three independent experiments done in duplicates.

### 4.8. Superoxide Dismutase Evaluation

The SOD activity was measured using commercially available kit (SOD activity Enzo Life Sciences, Farmingdale, NY, USA). The principle of the method is based on the ability of SOD to neutralize superoxide ions created by the xanthine/xanthine oxidase system and subsequently inhibits the reduction of WST-1 (water soluble tetrazolium salt) to WST-1 formazan. Briefly, 3T3 fibroblasts (5 × 10^6^ in six-well plate), obtained 24 h after oxidative stress induction, were washed with ice-cold 1× PBS, and lysed as described in kit protocol. The supernatant of each sample was collected, and the total SOD activity was assayed spectrophotometrically at 450 nm. SOD concentration, expressed in units per milligram of protein, was determined using the SOD standard curve.

### 4.9. Glutathione Evaluation

To assess the total level of glutathione, the 3T3 cells (5 × 10^6^ in six-well plate), obtained 24 h after oxidative stress induction, were washed with ice-cold 1× PBS, removed, resuspended in PBS and centrifuged (3000× *g* at 4 °C) twice for 5 min. After this process, the suspension obtained was then diluted in 50% trichloroacetic acid (Vetec, São Paulo, SP, Brazil) and centrifuged during 15 min (3000× *g* at 4 °C). After, the cell supernatant was diluted with the same volume 0.4 M Tris buffer contained 0.01 M dithiobisnitrobenzoic (Sigma-Aldrich, São Paulo, SP, Brazil). The material was read at 412 nm. The results were expressed as nmol/10^6^ cells.

### 4.10. Malonaldehyde Levels

To assess lipid peroxidation, malonaldehyde (MDA) production was measured with thiobarbituric acid reaction. Briefly, the cells under the same conditions as described above were triturated in 20 mM Tris-HCl buffer and centrifuged during 15 min (3000× *g* at 4 °C). The chromogenic reagent (10.3 mM 1-methyl-2-phenylindole in acetonitrile, 3:1 *v/v*), and a 37% solution of HCl were dropped to each supernatant sample (150 mL). The samples were kept at 45 °C for 40 min, and then were centrifuged (15 min; 3000× *g*; 4 °C). The absorbance was measured at 586 nm, and the results were expressed as nmol/10^6^ cells.

### 4.11. Antioxidant Activity

Four assays were performed to analyze the antioxidant activity of the sulfated polysaccharides obtained: total antioxidant capacity, hydroxyl radical scavenging, superoxide radical scavenging, and ferric chelating, as previously described [11,23].

#### 4.11.1. Determination of Total Antioxidant Capacity

This assay is based on the reduction of Mo (VI) to Mo (V) by sulfated polysaccharides and subsequent formation of a green phosphate/Mo(V) complex at acid pH. Tubes containing sulfated polysaccharides and reagent solution (0.6 M sulfuric acid, 28 mM sodium phosphate and 4 mM ammonium molybdate) were incubated at 95 °C for 90 min. After the mixture had cooled to room temperature, the absorbance of each solution was measured at 695 nm against a blank. Total antioxidant capacity was expressed as ascorbic acid equivalent.

#### 4.11.2. Hydroxyl Radical Scavenging Activity Assay

The scavenging activity of seaweed polysaccharides against the hydroxyl radical was investigated using Fenton’s reaction (Fe^2+^ + H_2_O_2_ → Fe^3+^ + OH^−^ + OH). These results were expressed as inhibition rate. Hydroxyl radicals were generated using 3 mL sodium phosphate buffer (150 mM, pH 7.4), which contained 10 mM FeSO_4_.7H_2_O, 10 mM EDTA, 2 mM sodium salicylate, 30% H_2_O_2_ (200 mL) and varying polysaccharide concentrations. In the control, sodium phosphate buffer replaced H_2_O_2_. The solutions were incubated at 37 °C for 1 h, and the presence of the hydroxyl radical was detected by monitoring absorbance at 510 nm. Gallic acid was used as positive control.

#### 4.11.3. Superoxide Radical Scavenging Activity Assay

This assay was based on the capacity of sulfated polysaccharides to inhibit the photochemical reduction of NBT in the riboflavin–light–NBT system. Each 3 mL of reaction mixture contained 50 mM phosphate buffer (pH 7.8), 13 mM methionine, 2 mM riboflavin, 100 mM EDTA, NBT (75 mM) and 1 mL sample solution. After the production of blue formazan, the increase in absorbance at 560 nm after 10 min illumination from a fluorescent lamp was determined. The entire reaction assembly was enclosed in a box lined with aluminum foil. Identical tubes with the reaction mixture were kept in the dark and served as blanks. Gallic acid was used as positive control.

#### 4.11.4. Ferric Chelating

The ferrous ion chelating ability of samples was investigated using the following methodology: sulfated polysaccharides at different concentrations were applied with the reaction mixture, which contained FeCl_2_ (0.05 mL, 2 mM) and ferrozine (0.2 mL, 5 mM). The mixture was shaken and incubated for 10 min at room temperature and absorbance of the mixture was measured at 562 nm against a blank. EDTA was used as positive control.

#### 4.11.5. Cupric Chelating

Pyrocatechol violet, the reagent used in this assay, has the ability to associate with certain cations such as aluminum, copper, bismuth, and thorium. In the presence of chelating agents this combination is not formed, resulting in decreased staining. This reduction thus allows the estimation of the chelating activity of the copper ion from the fraction from *U. flabellum*. The test is performed in 96-well microplates with a reaction mixture containing different concentrations of samples (0.1–20 mg/mL), pyrocatechol violet (4 mM), and copper II sulfate pentahydrate (50 mg/mL). All wells were homogenized with the aid of a micropipette and the solution absorbance was measured at 632 nm. The ability of the samples in chelating the copper ion was calculated using the following equation:

(Absorbance of blank) − (Absorbance of the sample)/(Absorbance of the blank) × 100

### 4.12. Electrophoresis in Agarose Gel

Agarose gel electrophoresis of the acidic polysaccharides was performed in 0.6% agarose gel (7.5 cm × 10 cm × 0.2 cm thick) prepared in 0.05 M 1.3-diaminopropane acetate buffer pH 9.0, as previously described [15]. Aliquots of the polysaccharides (about 50 μg) were applied to the gel and subjected to electrophoresis. The gel was fixed with 0.1% cetyltrimethylammonium bromide solution for 2 h, dried, and stained for 15 min with 0.1% toluidine blue in 1% acetic acid in 50% ethanol. The gel was then destained with the same solution without the dye.

### 4.13. Statistical Analysis

All data were expressed as mean ± standard deviation of three observation (*n* = 3). Statistical analysis was done by one-way analysis of variance (ANOVA) followed by the Turkey-Kramer test. All tests were conducted on the SigmaPlot^®^ (Systat software, San Jose, CA, USA). In all cases, statistical significance was set at *p* < 0.05.

## 5. Conclusions

We obtained six antioxidant sulfated polysaccharide-rich fractions from the green alga *U. flabellum.* These fractions mainly comprised heterogalactans, and were designated UF-0.3, UF-0.5, UF-0.6, UF-0.7, UF-1.0, and UF-2.0. None of the fractions exhibited cytotoxicity or inhibited 3T3 fibroblast proliferation. The four fractions that were assessed protected the cells from oxidative damage. UF-2.0 was the most effective in reducing oxidative stress when the cells were exposed to CuSO4; this can be attributed to the copper chelating ability of the fraction. The most effective fraction in mitigating oxidative stress in the presence of FeSO4 was UF-0.7. However, this effectiveness cannot be attributed solely to the ability of the fractions to chelate iron.

Our aims for future studies include identification of other mechanisms involved in mitigation of oxidative stress caused by compounds of iron and the role of sulfated polysaccharides in such mitigation. In addition, as UF-0.7 showed notable protective activity both in the presence of copper and iron, we intend to assess its effect in vivo to identify its role as a putative drug to be used for the treatment of diseases associated with elevated levels of copper and/or iron.

## Figures and Tables

**Table 1:** Chemical composition of polysaccharides extracted from the *U. flabellum*; Gal: galactose; Xyl: xylose; Man: mannose; Gluc: glucose; -Traces; n.d—not detected.

Sulfated Polysaccharides	Yield ^a^ (%)	Sulfate (%)	Protein (%)	Molar Ratio			
				Gal	Glu	Man	Xyl
UF-0.3	4.20	7.3 ± 0.8	4.9 ± 0.8	1.0	1.1	n.d	n.d
UF-0.5	36.9	18.7 ± 0.8	-	1.0	0.5	-	-
UF-0.6	9.9	10.7 ± 1.2	n.d	1.0	0.4	0.2	-
UF-0.7	17.7	17.0 ± 1.8	-	1.0	0.4	0.2	-
UF-1.0	11.5	9.8 ± 0.7	5.6 ± 0.8	1.0	0.7	0.7	0.3
UF-2.0	19.7	21.5 ± 1.3	n.d	1.0	1.0	1.0	n.d

^a^ All polysaccharides obtained by acetone precipitation were dried and weighed and total mass of each sample corresponded to 100%.

**Table 2:** Evaluation of the protective effect of the polysaccharides on the 3T3 cells exposed to the two models of oxidative damage.

**Effect of Sulfated Polysaccharides (1 mg/mL) on Total Glutathione (GSH) (mmol/10^6^ Cell) Levels of 3T3 Cells Exposed to Oxidative Damage**				
		FeSO_4_		CuSO_4_
NC	6.78 ± 2.21		7.01 ± 2.11	
PC		2.37 ± 0.60 ^a^		2.47 ± 0.43 ^a^
UF-0.5		3.63 ± 0.61 ^b^		4.71 ± 1.01 ^c^
UF-0.6		4.18 ± 0.98 ^c^		4.52 ± 0.93 ^c^
UF-0.7		4.75 ± 0.78 ^c^		4.89 ± 1.12 ^c^
UF-2.0		4.76 ± 1.08 ^c^		5.77 ± 1.08 ^c^
**Effect of Sulfated Polysaccharides (1 mg/mL) on Malondialdehyde (MDA) (mmol/10^6^ cell) Levels of 3T3 Cells Exposed to Oxidative Damage**				
		FeSO_4_		CuSO_4_
NC	250.1 ± 21.1		217.2 ± 41.7	
PC		2123.8 ± 99.7 ^a^		2312.1 ± 103.5 ^a^
UF-0.5		1542.5 ± 123.1 ^b^		1428.5 ± 111.2 ^b^
UF-0.6		1488.1 ± 209.2 ^b^		15012 ± 93.9 ^b^
UF-0.7		1107.0 ± 89.1 ^c^		1001 ± 101.1 ^c^
UF-2.0		1083.0 ± 56.3 ^c^		987 ± 95.1 ^c^
**Effect of Sulfated Polysaccharides (1 mg/mL) on Total Superoxide Dismutase (SOD) Levels (U/mg Protein) of 3T3 Cells Exposed to Oxidative Damage**				
		FeSO_4_		CuSO_4_
NC	33.5 ± 1.1		31.2 ± 0.9 ^a^	
PC		8.2 ± 2.1 ^a^		10.2 ± 1.1 ^a^
UF-0.5		11.8 ± 2.1 ^b^		13.9 ± 1.5 ^b^
UF-0.6		10.9 ± 1.0 ^b^		14.7 ± 1.2 ^b^
UF-0.7		14.2 ± 0.4 ^b^		20.8 ± 1.5 ^c^
UF-2.0		15.8 ± 0.7 ^b^		20.9 ± 0.8 ^c^

PC—positive control-culture medium with fetal bovine serum FeSO_4_ or CuSO_4_ and ascorbate. ^a^ *p* < 0.001 vs. NC; ^b^ *p* < 0.05; ^c^ *p* < 0.001 vs. PC.
